# Immune thrombocytopenic purpura caused by the over‐the‐counter weight supplement Root of Tejocote (*Crataegus* species)

**DOI:** 10.1002/ccr3.2804

**Published:** 2020-03-20

**Authors:** Hussam Alhasson, Eugene Muchnik

**Affiliations:** ^1^ Department of Internal Medicine Rochester Regional Health‐Unity Hospital Rochester New York; ^2^ Department of Hematology and Oncology University of Rochester Rochester New York

**Keywords:** Alipotec, immune thrombocytopenic purpura, root of tejocote, thrombocytopenia

## Abstract

Over‐the‐counter supplements, such as Alipotec which often purported to amplify weight loss and readily available in naturopathic shops, can have clinically significant patient outcomes including severe cytopenia, and even inducing immune thrombocytopenic purpura.

## Graphical abstract

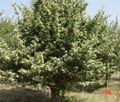

## 1 INTRODUCTION

Root of Tejocote is often purported to amplify weight loss and marketed under the brand name Alipotec^®^. This agent is readily available in naturopathic shops and online for purchase without any US Food and Drug Administration (FDA) indication for medicinal merit. We present the first known case in the literature of a patient who consumed Alipotec^®^ and developed immune thrombocytopenia purpura (ITP).

## 2 CASE PRESENTATION

A 51‐year‐old female with the history of hypothyroidism presented to the primary care office with complaint of generalized malaise for roughly 1 month duration. Hemogram revealed new thrombocytopenia with a platelet count of 62 × 10^3^/μL. The remainder of her complete blood count (CBC) showed a normal hemoglobin of 12.7 g/dL and white blood cell count (WBC) of 4.7/μL. Her most recent CBC from 6 months prior showed a hemoglobin of 12.8 g/dL, WBC of 6.3/μL, and platelet count of 406 × 10^3^/μL. Repeat CBC, ordered by the primary care physician 72 hours later, showed worsening thrombocytopenia with platelet count of 43 × 10^3^/μL, and the patient was instructed to seek care in the local emergency department (ED). She presented to the ED the following day, and her platelet count had decreased further to 25 × 10^3^/μL. After discussion with the on‐call hematologist, the patient was administered 1 mg/kg of prednisone (60 mg) and discharged on this dose with an outpatient hematology appointment.

The patient was seen in the hematology clinic 5 days after discharge. During her initial visit, she had no active bruising or bleeding and denied any prior bleeding tendency. She denied any antecedent coryza, fever, or pharyngitis. Her medications included levothyroxine, an over‐the‐counter multi‐vitamin, and a weight loss supplement, Alipotec^®^ (Tejocote). She drank alcohol socially. She denied any nicotine use or illicit drug use. She did not have a family history of blood diathesis. She works as a professional horse jockey. On physical examination, she had a small ecchymosis on her left elbow. No hepatosplenomegaly or lymphadenopathy was identified. The patient did not exhibit any overt gingival bleeding or petechial rash. Testing for Hepatitis B and C, HIV, Helicobacter Pylori serology, and heterophile antibodies was negative. TSH and T4 were checked and were 0.2 and 7.6, respectively. ESR, CRP, and ANA were negative.

A blood smear from her initial CBC in the ED was analyzed in the office and did not reveal pseudothrombocytopenia; there was no evidence of platelet clumping or platelet satellitism. Rare large platelets were appreciated on the index smear. There was no evidence of dysplasia in the red blood cell (RBC) or WBC cell lineages. Her platelet count was rechecked in the office and improved to 365 × 10^3^/μL on the 60 mg of prednisone. The patient was presumed to have ITP. Upon further questioning in the office, the patient admitted to starting a new weight loss supplement, Alipotec 6 weeks prior to ED evaluation in an attempt to lose weight for her aforementioned employment as a professional horse jockey.

The patient was started on a prednisone taper which was gradually reduced by 10 mg weekly. Four weeks later, at a dose of 20 mg of prednisone, her platelet count dropped from 266‐14 × 10^3^/μL. Prednisone was increased back to 60 mg. Despite this increase in steroids, her platelet count increased to only 62 × 10^3^/μL after 1 week.

Discussion was had about pursuing a bone marrow biopsy to exclude less common entities such amegakaryocytic thrombocytopenia, aplastic anemia, myelodysplastic syndrome, and overt leukemia, but the patient declined and continued on prednisone. Unfortunately, the patient had poor tolerance to steroids with frequent headaches, labile mood, and insomnia. Her platelet count continued to fluctuate between 50 and 87 × 10^3^/μL on glucocorticoids. Given the intolerance to prednisone and the relatively modest increase in her platelet count, a bone marrow biopsy was again suggested but patient declined.

Instead, the patient was next started on the Thrombopoietin Receptor Agonists (TPO‐RAs), Eltrombopag, at 50 mg daily. Unfortunately, after a singular dose, she developed nausea and vomiting and refused further treatment with the agent. At this time, both the patient and the treating hematologist began suspecting that her Alipotec^®^ may be the cause for her thrombocytopenia. She was advised to discontinue it, but she was reluctant to do so given the weight loss she was experiencing on the supplement.

At her next hematology appointment, which was 9 weeks from her index hematology visit, the patient was given the option to proceed with curative intent splenectomy or Rituxan treatment. The patient was informed that these remedies would only be attempted if she would agree to a diagnostic bone marrow biopsy. The patient again refused invasive testing.

A hemogram the following week, surprisingly, revealed a platelet count of 342x10^3^/μL, despite the patient admitting to self‐reducing her prednisone from 40 to 10 mg. Upon further inquiry, she also admitted to stopping her Alipotec in hopes that the over‐the‐counter supplement was the cause of the ITP. Her platelet count the following 3 weeks continued to remain over 200 × 10^3^/μL, despite the patient self‐discontinuing all prednisone products.

Four weeks after stopping the Alipotec^®^ and prednisone, her platelet count decreased to 46 × 10^3^/μL. She confided that she resumed the Alipotec in preparation to a critical horse race, 4 days prior to the blood draw. She refused resuming any steroid products, and she again discontinued the offending agent once she was informed of her recurrent thrombocytopenia. She had a repeat hemogram the following week after discontinuing the Alipotec^®^, which again showed correction of her thrombocytopenia with platelet count of 336 × 10^3^/μL. Repeated CBC 1 month later off any treatment continued to show normal platelet counts, and the patient stated she had completely stopped the Alipotec.

## 3 DISCUSSION

Thrombocytopenia is defined as a platelet count below the lower limit of normal (<150 × 10^3^/μL). While there are many etiologies of thrombocytopenia, including peripheral destruction, decreased bone marrow production, and increased splenic sequestration, a frequent entity for severe isolated thrombocytopenia is ITP. ITP, formerly known as idiopathic thrombocytopenia purpura, is an acquired autoimmune condition where IgG type antibodies are directed against platelet membrane glycoproteins IIb‐IIIa leading to peripheral destruction.^1^ While antibody and complement mediated platelet destruction by macrophage clearance in the reticuloendothelial system is felt to best explain the disease process, the pathophysiology remains incompletely understood.^2^ In fact, up to 50% of patients with ITP will not have detectable autoantibodies and such antibody testing is not felt to be part of standard diagnostic workup.^3^ The lack of identifiable antibody formation might be due to a second mechanism of platelet clearance, namely, that platelet destruction is also mediated by cytotoxic T cells and cellular immunity rather than humoral immunity.^2^ Thrombocytopenia from ITP often manifests as mucocutaneous bleeding, petechial rash, spontaneous bruising, and rarely, intracranial hemorrhage.^4^

ITP in adults and children manifests differently, most notably for the high rates of spontaneous resolution in the pediatric population compared to the adult population.^5^ Adults with ITP have spontaneous resolution rates of only 9%, and therefore, often require prolonged immunosuppression.^6^ The cornerstone of ITP treatment remains glucocorticoids; administered either with daily prednisone at 1‐2 mg/kg for 1‐2 weeks followed by gradual taper or as high pulse dose of dexamethasone at 40 mg for 4‐5 days.^7^ Compared to intravenous immunoglobulin (IVIG), prednisone has demonstrated similar efficacy and is associated with lower cost and feasibility of administration.^8^ Despite the efficacy of glucocorticoids, adult patients uncommonly can be weaned off completely with high rates of disease relapse.^9^ As such, further management with TPO‐RAs, monoclonal antibody immune suppression treatment with rituximab, or splenectomy is often employed for long‐term control.^10^, ^11^

Many causes for ITP have been established, including non‐Hodgkin's lymphoma such as chronic lymphocytic leukemia (CLL), viral infections such as HIV, Hepatitis C, or infectious mononucleosis, medications such as Quinine, Trimethoprim‐Sulfamethoxazole, and Vancomycin, and autoimmune conditions including hypothyroidism, rheumatoid arthritis, and systemic lupus erythematosus.^3^, ^12^, ^13^, ^14^, ^15^, ^16^, ^17^ While the data on prescription drugs are robust with respect to thrombocytopenia, over‐the‐counter supplements, which are often not scrutinized by the FDA, may have unintended consequences that are not readily reported by the manufacturers.

**Figure 1:**
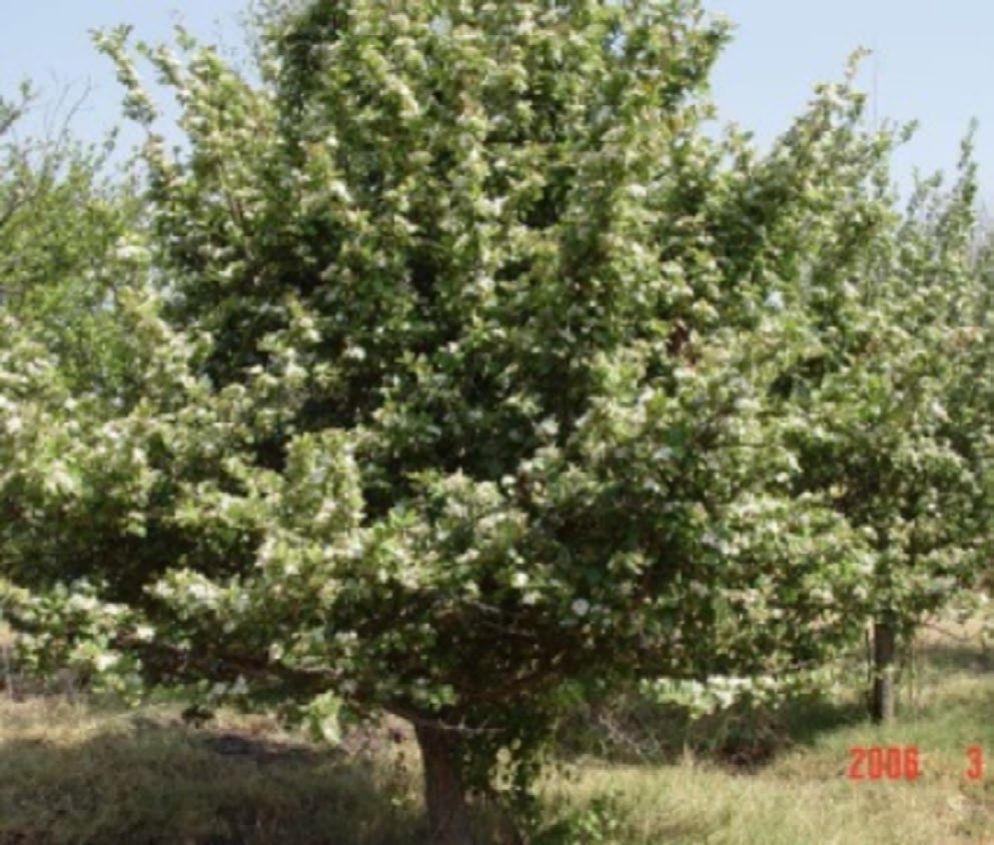
Tejocote Tree. Source Ref. 26

**Figure 2:**
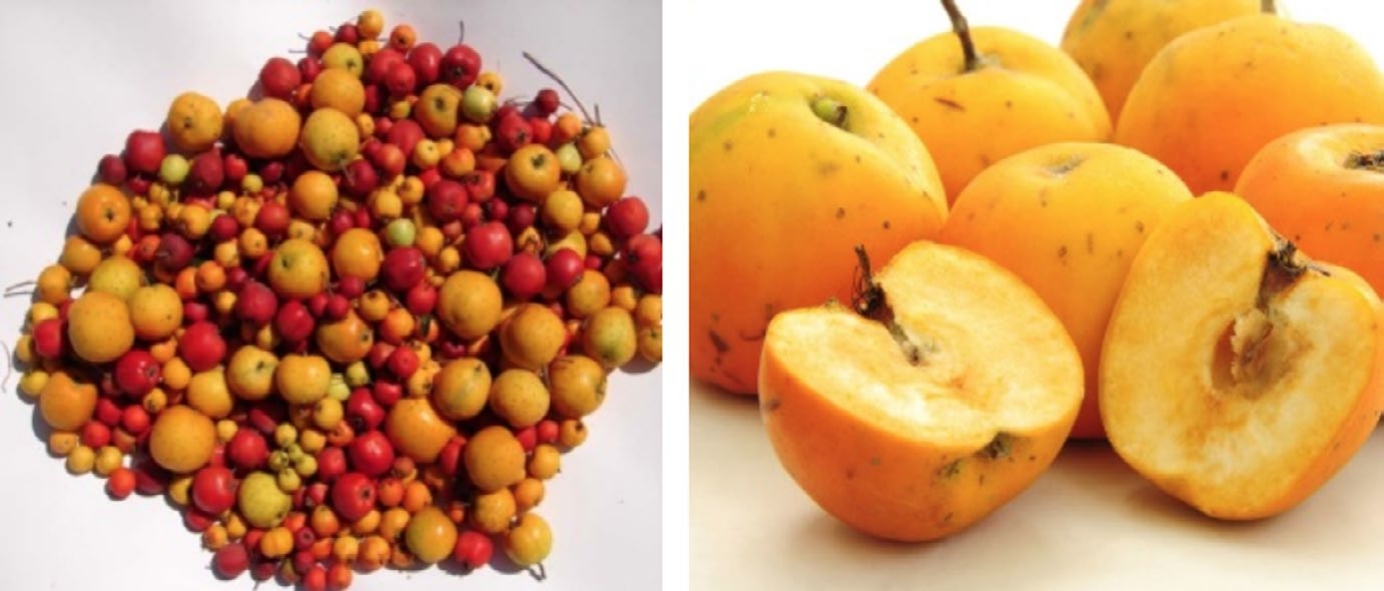
Tejocote fruit. Source Ref. 26

Tejocote (*Crataegus Species*) is a genus of fruit‐bearing Hawthorn trees (Figure 1) distributed mostly in Mexico and in Latin America.^18^ Thirteen species are identified and classified according to different shoot types, fruits, and flowers.^19^ Tejocote fruit (Figure 2) which are rich in Vitamin C, antioxidants, and pectin fibers are often collected for direct consumption and processed into jams, jelly, or sirup.^18^ Medicinally, *Crataegus* ingredients were historically reported to be used in treating diarrhea, cough suppression, and even managing kidney pain and cardiac ailments.^19^, ^20^

**Figure 3:**
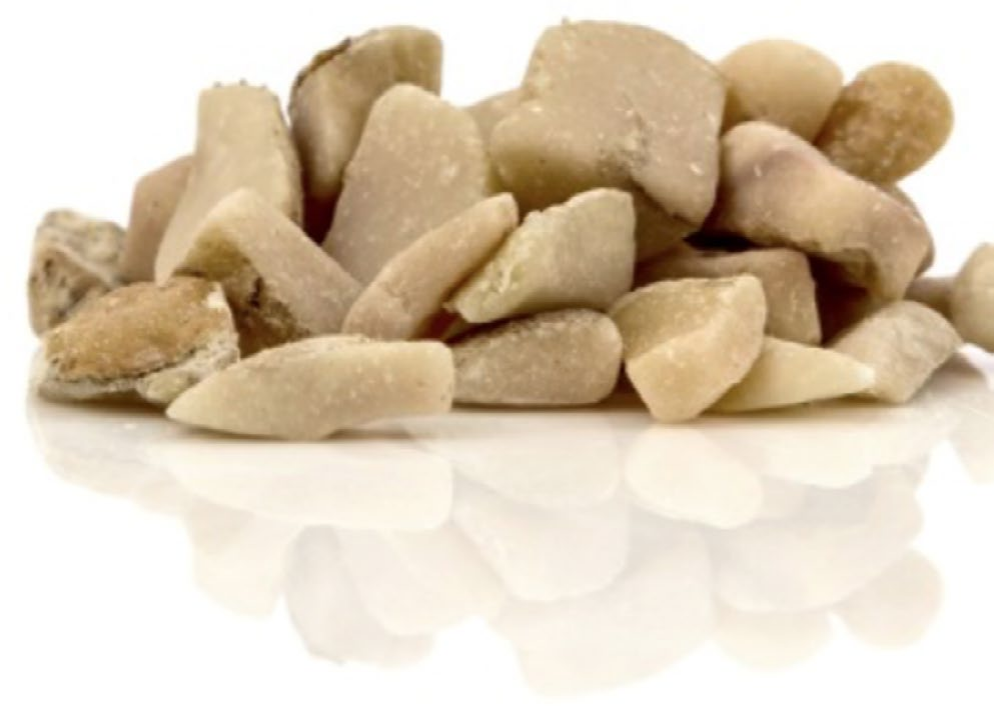
Alipotec supplement. Source Ref. 26

Alipotec (Figure 3) is a brand name product derived from Root of Tejocote. It is a non‐FDA approved over‐the‐counter supplement that is promoted as a weight loss remedy.One presumed mechanism for the weight loss is the high pectin content of the Tejocote root, which has been associated with early satiety.^21^ Side‐effects reported by the supplier include GI upset and myalgias, the former presumably from the high pectin content.^22^ Other side‐effects noted in the literature include possible cardiotoxicity and drug‐drug interactions, including false negative elevation in digoxin assay.^23^

**Graph 1:**
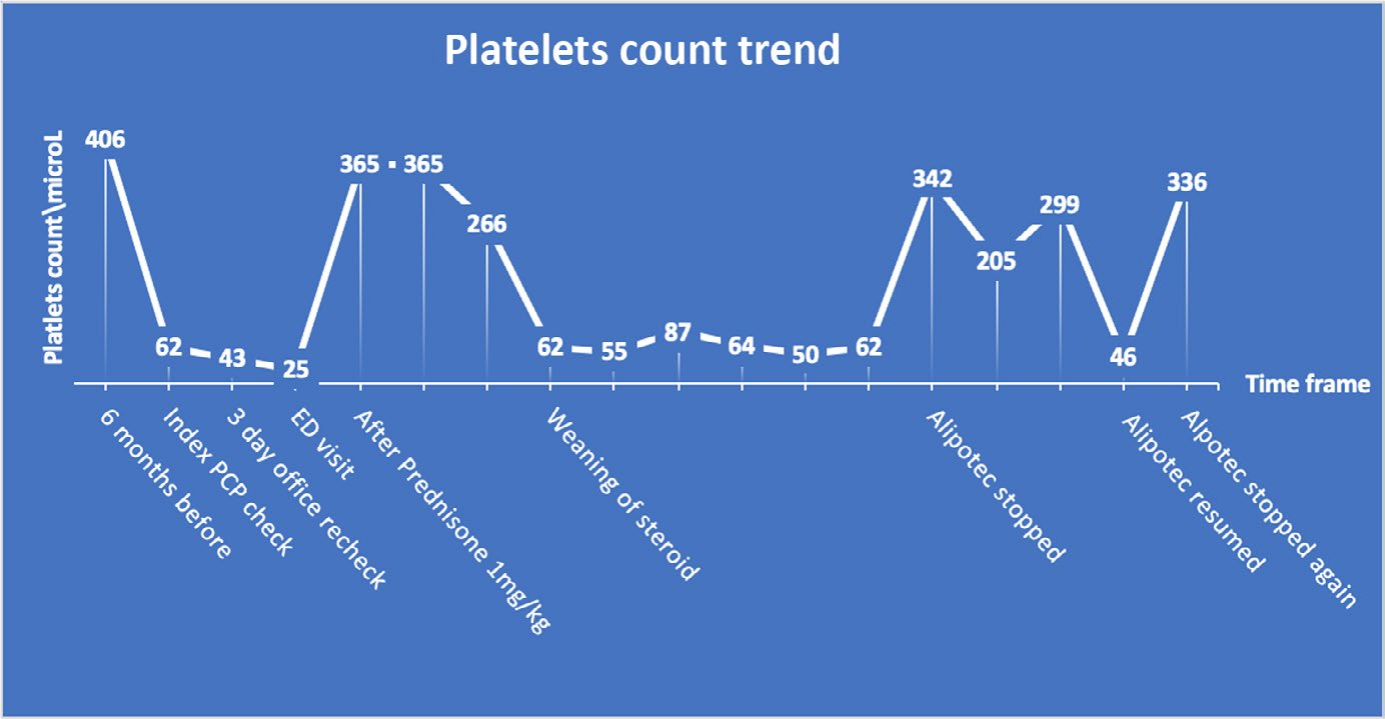
Platelet count trends during the course of the Immune thrombocytopenia (ITP) from diagnosis to resolution

ITP has established criteria to gauge treatment response.^24^ Following holding of the Alipotec, our patient had a complete response of her ITP, as defined by a platelet count of greater than 100 × 10^3^/μL measured on two occasions 7 days apart. At the time of writing this case, the patient has remained with a platelet count of greater than 300 000 over the past 12 months with her last platelet count of 415 × 10^3^/μL while remaining off the Alipotec (Graph 1).

In our patient, drugs‐dependent antibodies were not checked as negative does not rule out the diagnosis with low sensitivity.^25^ Also, our patient tested negative to HIV, HCV and had negative heterophile antibodies. Also, she denied temporal exposure to medications known to cause ITP including Quinine. She is a known hypothyroidism on Levothyroxine and had mildly low T4 and, however, her platelets count remained to fluctuate rapidly. Thyroid function remained mildly low upon recheck after her platelets consistently normalized. Inadvertently due to repeated use of Alipotec by patient, she demonstrated positive drug rechallenge which exhibit high quality evidence that her Alipotec use was responsible for her thrombocytopenia.

Thrombocytopenia is an unknown, but a possible serious, adverse effect of Alipotec that has never been reported in the literature. Our patient showed immediate, dramatic recovery of her platelet count following discontinuation of Alipotec^®^. The mechanism of how Alipotec causes presumed immune‐mediated thrombocytopenia is currently unknown.

## 4 CONCLUSION

Detailed history taking including medication and over‐the‐counter supplement use in patients with newly unexplained thrombocytopenia is essential. When there is high clinical suspicion that a nonprescription agent might be responsible for adverse events, such as ITP, these agents should be empirically held, and the patient's clinical status and laboratory evaluation should be reassessed.

## CONFLICT OF INTEREST

None declared.

## AUTHOR CONTRIBUTIONS

HA drafted and took the lead in writing the manuscript as well as created the graph and figures. EM revising manuscript critically for important intellectual content. HA and EM both contributed to and approved the final version of the manuscript.
